# Pattern of congestive heart failure in a Kenyan paediatric population

**DOI:** 10.5830/CVJA-2013-015

**Published:** 2013-06

**Authors:** Julius A Ogeng’o, Patrick M Gatonga, Beda O Olabu, Diana K Nyamweya, Dennis Ong’era

**Affiliations:** Department of Human Anatomy, University of Nairobi, Nairobi, Kenya; Department of Human Anatomy, University of Nairobi, Nairobi, Kenya; Department of Human Anatomy, University of Nairobi, Nairobi, Kenya; Department of Human Anatomy, University of Nairobi, Nairobi, Kenya; Department of Human Anatomy, University of Nairobi, Nairobi, Kenya

**Keywords:** heart failure, infections, paediatric, Kenya

## Abstract

**Background:**

Heart failure in children is a common cause of morbidity and mortality, with high socio-economic burden. Its pattern varies between countries but reports from Africa are few. The data are important to inform management and prevention strategies.

**Objective:**

To describe the pattern of congestive heart failure in a Kenyan paediatric population.

**Methods:**

This was a retrospective study done at Kenyatta National Hospital, Nairobi Kenya. Records of patients aged 12 years and younger admitted with a diagnosis of heart failure between January 2006 and December 2010 were examined for mode of diagnosis, age, gender, cause, treatment and outcome. Data were analysed using the Statistical Programme for Social Scientists version 16.0 for windows, and presented in tables, bar and pie charts.

**Results:**

One hundred and fifty-eight cases (91 male, 67 female) patients’ records were analysed. The mean age was 4.7 years, with a peak at 1–3 years. The male:female ratio was 1.4:1. All the cases were in New York Heart Association (NYHA) class II–IV. Evaluation of infants was based on the classification proposed by Ross *et al*. (1992). Diagnosis was made based on symptoms and signs combined with echocardiography (echo) and electrocardiography (ECG) (38%); echo alone (12.7%); ECG, echo and chest X-ray (CXR) (11.4%); and ECG alone (10.8%). The underlying cause was established on the basis of symptoms, signs, blood tests, CXR, echo and ECG results. Common causes were infection (22.8%), anaemia (17.1%), rheumatic heart disease (14.6%), congenital heart disease (13.3%), cardiomyopathy (7.6%), tuberculosis and human immunodeficiency virus (6.9% each); 77.9% of patients recovered, 13.9% after successful surgery, and 7.6% died.

**Conclusion:**

Congestive heart failure is not uncommon in the Kenyan paediatric population. It occurs mainly before five years of age, and affects boys more than girls. The majority are due to infection, anaemia, and rheumatic and congenital heart diseases. This differs from those in developed countries, where congenital heart disease and cardiomyopathy predominate. The majority of children usually recover. Prudent control of infection and correction of anaemia are recommended.

## Abstract

Congestive heart failure in a paediatric population is a common cause of morbidity and mortality and is a serious public health concern, with tremendous socio-economic impact.^1^,^2^ Its pattern varies between and within countries.^3^-^5^ In sub-Saharan Africa, studies mainly from Nigeria reveal that it accounts for 5.8–9.0% of emergency admissions to paediatric units.^2^,^4^,^5^ These causes vary between developed and developing countries, age and geographical location.^6^,^7^ These data are important in diagnosis, treatment, prognosis, control and prevention. Reports from eastern Africa are, however, scarce and altogether absent for Kenya. This study therefore investigated the pattern of congestive heart failure in a black Kenyan paediatric population.

## Methods

This was a retrospective study at Kenyatta National Hospital (KNH), Nairobi, Kenya, which is a 1 800-bed capacity teaching and eastern African regional referral centre. It receives about 30 000 paediatric in-patients a year, mainly from black Kenyans of middle to lower socio-economic class. This hospital has four paediatric cardiologists and 40 paediatric cardiology beds. Ethical approval for the study was granted by KNH/University of Nairobi Ethics and Research committee.

Records of patients aged 12 years and younger who were admitted to the hospital with heart failure according to New York Heart Association (NYHA) classification II–IV between January 2006 and December 2010 were retrieved from the hospital registry. In infants, diagnosis and classification was based on criteria proposed by Ross *et al*. (1992).^8^ Patients were divided into male and female gender. Each gender category was subsequently divided into infants (one year and below), and four age groups of three years each, starting at one year.

Subsequently, the records were examined for cause and sub-cause of heart failure based on clinical, echo, ECG, CXR and laboratory findings. The causes were divided into six categories, namely congenital heart disease (CHD), rheumatic heart disease (RHD), anaemia, infections, cardiomyopathy, and other. In the categories where there were more than 25 patients, they were further subdivided according to specific cause. Those cases in whom some data on the parameters above were missing were excluded from the study.

Data obtained were analysed using Statistical Programme for Social Scientists (SPSS) version 16.0 for windows, and presented in tables, bar and pie charts.

## Results

One hundred and sixty-five cases were retrieved. Seven were excluded from the study: four in whom age, and three in whom the cause of heart disease was not recorded. One hundred and fifty-eight cases (91 males, 67 females) were analysed.

**Fig. 1.**
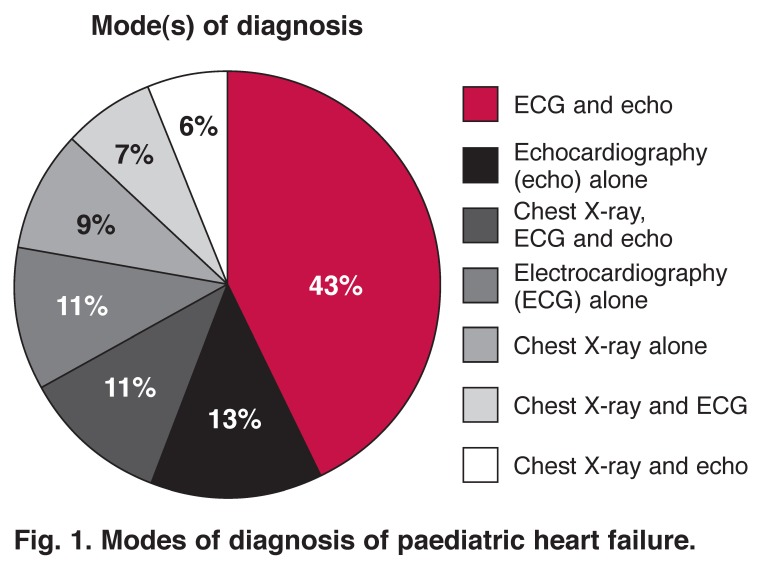
Modes of diagnosis of paediatric heart failure.

Diagnosis was made on the basis of symptoms and signs. In infants, these were feeding difficulties, increased fatiguability, tachynoea, intercostal retraction, dysponoea, grunting, tachycardia, gallop rhythm, cyanosis, rales and hepatomegaly. In older children, the symptoms and signs included exercise intolerance, somnolence, anorexia, tachypnoea, dyspnoea, orthopnoea, cough, wheezing, rales, gallop rhythm, oedema, hepatomegaly, and raised jugular venous pressure. The most common investigations used to complement clinical diagnosis were echocardiography combined with ECG (43.0%), and echo alone (12.6%). Echo, ECG and CXR were done in 11.4 %, and ECG alone in 10.8% (Fig. 1).

Routine laboratory tests done for all patients included total blood count (TBC), peripheral blood film (PBF), and urea, electrolytes and creatinine (U/E/Cs) levels. Specific tests done were brain natriuretic peptide (BNP) (12.6%), C-reactive protein (CRP) (11.4%), tumour necrotic factor (TNF) alpha (11.4%), human immunodeficiency virus (HIV) ELISA (13.8%), CD4 counts (13.8%), and blood cultures (13.3%). The Mantoux test was done in those suspected to have tuberculosis.

**Fig. 2.**
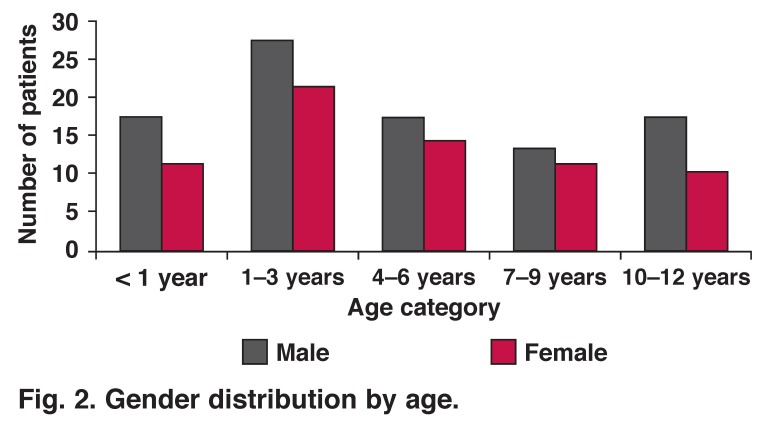
Gender distribution by age.

The mean age of these patients was 4.7 years, peaking at 1–3 years (range: 2 months – 12 years). The male:female ratio was 1.4:1, and the male predominance persisted through all age groups (Fig. 2).

The most common single cause was infections (22.8%). Among these, the infections recorded were pneumonia (50%), upper airway (19.4%), throat (16.7%), and urinary tract (13.9%) infections. Anaemia (17.1%) was the second most common cause. The anaemia was due to malaria (48.2%), helminthiasis (22.2%), malnutrition (14.8%), and sickle cell disease (14.8%). Other causes included rheumatic (14.6%) and congenital (13.3%) heart disease, cardiomyopathy (7.6%), tuberculosis and HIV (6.9%) (Table 1).

**Table 1:** Distribution Of Causes Among Congestive Heart Failure Paediatric Patients

	*Distribution*			
*Cause*	*Male*	*Female*	*Total*	*%*
Infections	20	16	36	22.8
Anaemia	17	10	27	17.1
Rheumatic heart disease	12	11	23	14.6
Congenital heart disease	10	11	21	13.3
Cardiomyopathy	5	7	12	7.6
Tuberculosis	5	6	11	6.9
HIV	8	3	11	6.9
Adenoid hypertrophy	2	1	3	1.9
Rickets	1	2	3	1.9
More than one cause	6	0	6	3.8
More than two causes	5	0	5	3.2
Total	91	67	158	100

Eleven (7.0%) of the cases had multiple causes; six more than one, and five more than two. In six cases (3.8%), HIV was combined with tuberculosis. In five cases (3.2%), there were more than two causes: three in which bacterial infection was combined with cardiomyopathy and HIV, and two in which there was malaria, anaemia and infection.

Treatment comprised appropriate correction of cause of heart failure, such as treatment of infection with antibiotics, blood on cause.11 Accordingly, age distribution is expected to vary between countries and centres.

## Discussion

Observations of the current study reveal that heart failure constitutes about 1:1 000 of paediatric hospital admissions. It is difficult to compare rates among hospitals due to different hospital sizes, paediatric age limit, diagnostic criteria, age distribution and profile of causes.

The modes of diagnosis combining clinical symptoms, signs and investigations are concordant with those practiced elsewhere.^9^,^10^ This implies that detection rates are comparable to those in other centres. Accordingly, any differences may result from other factors, probably related to age and cause.

The mean age at presentation was 4.7 years, comparable to the 3.7 ± 3.5 years reported from Nigeria.^2^ Notably, this is beyond infancy and is commensurate with observations that congenital defects constitute less than acquired causes of heart failure. In developed countries where congenital heart disease is the leading cause,^1^,^9^,^11^ the mean age is much lower. The mean age is also affected by one-year survival rates and is dependent on cause.^11^ Accordingly, age distribution is expected to vary between countries and centres.

The 1.4:1 male-to-female ratio recorded in the present study is comparable to the 1.5:1 for Nigeria,^2^ suggesting relative uniformity in gender-related factors affecting the distribution of congestive heart failure in a Kenyan paediatric population.

Infection was the leading cause of heart failure, followed by anaemia, and rheumatic and congenital heart diseases. This is at variance with literature reports from developed countries where most causes are congenital heart disease and cardiomyopathy.^1^,^3^ It however resembles results from Ibadan in Nigeria.^12^ Several other studies support variations in the leading causes of heart failure in children between developed and developing countries (Table 2). These variations may be due to differences in awareness of preventive measures and access to healthcare services.

**Table 2:** Causes Of Pediatric Heart Failure In Different Countries

*Author*	*Population*	*Top four causes (%)*
Adekambi *et al.* 2007[Bibr R05]	Nigerian	Anaemia (46), infection (29), anaemia + infection (11.5), CHD (10.5)
Massin *et al.* 2008^9^	Belgian	CHD (51.6), cardiomyopathy (19.4), RHD (10.5), pericardits (5.6)
Andrews *et al.* 2008^11^	United Kingdom	Cardiomyopathy (55.8), myocardits (19.6), arrythmia (5.6), anthracycline toxicity (4.0)
Borzouee *et al.* 2008^13^	Iranian	CHD (76), RHD (16.1), cardiomyopathy (4.0), other (3.8)
Current study	Kenyan	Infection (22.8), anaemia (17.1), rheumatic heart disease (14.6), congenital heart disease (13.3)

The infections most frequently associated with cardiac failure were pneumonia, upper respiratory and throat infections. This is concordant with reports from African countries where respiratory infection constitutes a significant cause of heart failure.^12^ Interestingly, the situation observed here resembles that which was obtained in the United Kingdom in the middle of the last century, when bronchitis, pneumonia and other respiratory infections were the most frequent causes of heart failure.^14^

A remarkable observation of the present study was that HIV in isolation or combination was associated with heart failure in 12.7% of the patients. This is commensurate with other studies, which reported that heart diseases such as pericarditis, myocarditis, cardiomyopathy and endocarditis were associated with HIV infection.^15^,^16^ The pathogenesis of heart muscle insufficiency probably involves the direct effects of the virus on the heart, an inflammatory response of the host myocardium to the virus, and the presence of auto-antibodies, as well as decreased immunity, which makes them more prone to infection.^17^ HIV is endemic in Kenya, and its high association with heart failure suggests that it should always be considered an important differential diagnosis, and that control of the disease is important in reducing heart failure.

Tuberculosis in HIV-negative patients was associated with heart failure in 6.9% of cases. This appears in tandem with increasing reports of tuberculous pericarditis and myocarditis with no evidence of HIV, and disseminated TB.^18^ Accordingly, as suggested before, myocardial involvement should be suspected as a cause of congestive cardiac failure in any patient with features of TB.^19^ Indeed, myocardial TB is well recognised and there are cases where cardiac TB presents with congestive cardiac failure.^20^

Tuberculosis is a common problem in Kenya. Its association with congestive heart failure is important for two reasons. Firstly, patients with TB should be monitored for cardiac involvement. Secondly, in heart failure patients, TB should be considered an important differential diagnosis.

Anaemia was the second most frequent cause of heart failure, affecting 17.1% of the children, again in sharp contrast with reports from developed countries (Table 2). This is, however, lower than the 28–46% reported from Nigeria.^5^,^12^ The contrast between European and African countries is concordant with the suggestion that causes of congestive heart failure in a Kenyan paediatric population depend on the stage of epidemiological transaction. The anaemia, similar to literature reports,^21^ was multifactorial, being caused by malaria, intestinal helminths, poor nutritional status, and haemoglobinopathy. These imply that a multi-prong approach to the control of anaemia constitutes a major step in mitigating heart failure.

Rheumatic heart disease is highly prevalent in Kenya, causing 32% of adult heart failure.^22^,^23^ In the current study, it constituted 14.6% of heart failure. It was notably higher than the 1% reported in Nigeria.^5^,^12^ This implies that control of rheumatic heart disease, for example, by prudent treatment of throat infections would substantially reduce congestive heart failure in a Kenyan paediatric population due to acquired causes. Pertinent to this suggestion are reports from developed countries indicating that RHD is no longer a significant cause of CCF.^1^,^11^

Congenital heart disease is the most important cause of infant heart failure in developed countries.^1^,^3^ In the current study, it ranks fourth but constitutes 13.3%, slightly higher than the 10.5% reported in a Nigerian study.^5^ This is in tandem with reports that CHD are common in Kenya,^24^ and indicates that it already constitutes a significant cause of heart failure.

Cardiomyopathy is the major cause of heart failure among children with normal hearts in developed countries.^1^,^9^,^11^ In the current study, it constituted 7.6%. This, while lower than the figures reported for developed countries, is higher than implied in reports from another African country in which it is not listed among the causes of heart failure.^5^,^12^ The other causes, namely adenoids and rickets, are also concordant with literature reports.^25^,^26^

The treatment modalities provided in KNH are in tandem with conventional practice.^10^ Mortality rate in this series was 7.7%. This is much lower than the 24% reported in Nigeria^12^ and 14% in Belgium.^9^ Outcomes of heart failure are difficult to compare because of different aetiological factors and accessibility to healthcare facilities. For example, in developed countries, most babies with CHD receive early surgical intervention,^27^ while in Kenya, a significant number may miss the opportunity to have optimal surgical care.^24^ Notably, however, the observation that cardiomyopathy which is known to have a relatively poor outcome,^28^ constitutes only a small proportion of cases, may partly explain the comparatively low mortality rate. Indeed, mortality rates have been reported to depend on the cause.^9^ This implies that with control of infection, the outcome of congestive cardiac failure may improve.

## Conclusion

Congestive heart failure is not uncommon in the Kenyan paediatric population. It occurs mainly before five years of age years and affects boys more than girls. The majority are due to infection, anaemia, and rheumatic and congenital heart diseases. This differs from those in developed countries, where congenital heart disease and cardiomyopathy predominate. The majority of children usually recover. Prudent control of infection and correction of anaemia are recommended.

## References

[1] Hsu DT, Pearson GD (2009). Heart failure in children. Part 1: History. Etiology and pathophysiology.. Circulation: Heart Failure.

[2] Oyedeji AO, Oluwayemi IO, Okeniyi JA, Fadero FF (2010). Heart failure in Nigerian children.. Cardiology.

[3] Venugopalan P, Agarwal AK, Akinbami FO, El Nour JB, Subramanyan R (1998). Improved diagnosis of heart failure in children.. Int J Cardiol.

[4] Lagunju IA, Omokhodion SI (2003). Childhood heart failure in Ibadan.. West Afr J Med.

[R05] Adekambi AF, Ongunlesi TA, Olowu AO, Fetuga MB (2007). Current trends in the prevalence and aetiology of childhood congestive cardiac failure in Sagamu.. J Trop Pediatr.

[6] Sharma M, Nair MNG, Jatana SK, Shahi BN (2003). Congestive heart failure in infants and children.. Med J Armed Forces Ind.

[7] James N, Smith M (2005). Treatment of heart failure in Children.. Curr Paediatr.

[8] Ross RD, Bollinger RO, Pinsky WW (1992). Grading the severity of congestive heart failure in infants.. Pediatr Cardiol.

[9] Massin MM, Astadicko I, Dessy H (2008). Epidemiology of heart failure in a tertiary pediatric centre.. Clin Cardiol.

[10] Madriago E, Silberbach M (2010). Heart failure in Infants.. Paediatr Rev.

[11] Andrews RE, Fenton MJ, Ridout DA, Burch M (2008). New onset heart failure due to heart muscle disease in childhood: a prospective study in the United Kingdom and Ireland.. Circulation.

[12] Omokhodion SI, Lagunju IA (2005). Childhood heart failure in Ibadan.. West Afr J Med.

[13] Borzouee M, Jannati M (2008). Distribution and characteristics of the heart disease in pediatric age group in southern Iran.. Iran Cardiovas Res J.

[14] Flint JF (1954). The factor of infection in heart failure.. Br Med J.

[15] Braun K, Izak G (1955). Acute pulmonary infection and cardiac failure in chronic emphysema.. Am Heart J.

[16] Sudano I, Spieke L, Noll G, Corti R, Weber R, Luscher TF (2006). Cardiovascular disease in HIV infection.. Am Heart J.

[17] Gopal M, Bhaskaran A, Khalife WJ, Barbagelata A (2009). Heart disease in patients with HIV/AIDS – An emerging clinical problem.. Curr Cardiol Rev.

[18] Afzal A, Koehane M, Keeley E, Borzak S, Callender CW, Iannuzzi M (2000). Myocarditis and pericarditis with tampande associa ted with disseminated tuberculosis.. Can J Cardiol.

[19] Agarwal R, Malhota P, Awasthi A, Kakkar N, Gupta D (2005). Tuberculous dilated cardiomyopathy an underrecognised sentity?. BMC Inf Dis.

[20] Brar R, Prasad A, Kumar A, Bagai M, Malhotra M (2010). Myocardial tuberculosis presenting with congestive heart failure and pulmonary venous occlusion.. Eur J Radiol.

[21] Crawley J (2004). Reducing the burden of Anaemia in infants and young children in malaria – endemic countries of Africa: from evidence to Action.. Am J Trop Med Hyg.

[22] Anabwani GM, Bonhoeffer P (1996). Prevalence of Heart disease in school children in rural Kenya using color flow echocardiography.. East Afr Med J.

[23] Oyoo GO, Ogolla EN (1999). Clinical and sociodemographic aspects of congestive heart failure at Kenyatta National Hospital.. East Afr Med J.

[24] Awori MN, Ogendo SW, Gitome SW, Onguti SK, Obonyo NG (2007). Management pathway for congenital heart disease at Kenyatta National hospital, Nairobi.. East Afr Med J.

[25] Maiya S, Sullivan I, Allgrove J, Yates R, Malone M, Brain C (2008). Hypocalcaemia and vitamin D deficiency: an important but preventable cause of life threatening infant heart failure.. Heart.

[26] Brown J, Munez S, Rusell M, Spurney C (2009). Hypocalcemic rickets and dilated cardiomyopathy: Case reports and review of literature.. Pediatr Cardiol.

[27] Kay JD, Colan SD, Graham TP (2001). Congestive heart failure in paediatric patients.. Am Heart J.

[28] Arola A, Jokinen E, Ruuskanen O, Saraste M, Pesonen E, Kuusela AL (1997). Epidemiology of Idiopathic cardiomyopathies in children and adolescents. A nation wide study in Finland.. Am J Epidemiol.

